# Diagnostic Performance of [^18^F]FDG PET in Staging Grade 1–2, Estrogen Receptor Positive Breast Cancer

**DOI:** 10.3390/diagnostics11111954

**Published:** 2021-10-21

**Authors:** Ramsha Iqbal, Lemonitsa H. Mammatas, Tuba Aras, Wouter V. Vogel, Tim van de Brug, Daniela E. Oprea-Lager, Henk M. W. Verheul, Otto S. Hoekstra, Ronald Boellaard, Catharina W. Menke-van der Houven van Oordt

**Affiliations:** 1 Department of Medical Oncology, Cancer Center Amsterdam, Amsterdam UMC, Vrije Universiteit Amsterdam, 1081 HV Amsterdam, The Netherlands; t.aras@student.vu.nl; 2 Department of Medical Oncology, Reinier de Graaf Gasthuis, 2625 AD Delft, The Netherlands; l.mammatas@rdgg.nl; 3 Department of Nuclear Medicine and Department of Radiation Oncology, The Netherlands Cancer Institute—Antoni van Leeuwenhoek, 1066 CX Amsterdam, The Netherlands; w.vogel@nki.nl; 4 Department of Epidemiology and Data Science, Amsterdam Public Health Research Institute, Amsterdam UMC, Vrije Universiteit Amsterdam, 1081 HV Amsterdam, The Netherlands; t.vandebrug@amsterdamumc.nl; 5 Department of Radiology and Nuclear Medicine, Cancer Center Amsterdam, Amsterdam UMC, Vrije Universiteit Amsterdam, 1081 HV Amsterdam, The Netherlands; d.oprea-lager@amsterdamumc.nl (D.E.O.-L.); os.hoekstra@amsterdamumc.nl (O.S.H.); r.boellaard@amsterdamumc.nl (R.B.); 6 Department of Medical Oncology, Radboud University Medical Center, 6525 GA Nijmegen, The Netherlands; Henk.Verheul@radboudumc.nl

**Keywords:** positron emission tomography (PET), [^18^F]FDG, breast cancer, estrogen receptor, staging

## Abstract

Positron emission tomography using [^18^F]fluorodeoxyglucose (FDG PET) potentially underperforms for staging of patients with grade 1–2 estrogen receptor positive (ER+) breast cancer. The aim of this study was to retrospectively investigate the diagnostic accuracy of FDG PET in this patient population. Suspect tumor lesions detected on conventional imaging and FDG PET were confirmed with pathology or follow up. PET-positive lesions were (semi)quantified with standardized uptake values (SUV) and these were correlated with various pathological features, including the histological subtype. Pre-operative imaging detected 155 pathologically verified lesions (in 74 patients). A total of 115/155 (74.2%) lesions identified on FDG PET were classified as true positive, i.e., malignant (in 67 patients) and 17/155 (10.8%) lesions as false positive, i.e., benign (in 9 patients); 7/155 (4.5%) as false negative (in 7 patients) and 16/155 (10.3%) as true negative (in 14 patients). FDG PET incorrectly staged 16/70 (22.9%) patients. The FDG uptake correlated with histological subtype, showing higher uptake in ductal carcinoma, compared to lobular carcinoma (*p* < 0.05). Conclusion: Within this study, FDG PET inadequately staged 22.9% of grade 1–2, ER + BC cases. Incorrect staging can lead to inappropriate treatment choices, potentially affecting survival and quality of life. Prospective studies investigating novel radiotracers are urgently needed.

## 1. Introduction

Breast cancer (BC) is the most frequently diagnosed malignancy among women worldwide [1]. In the Netherlands, 16,000 women are newly diagnosed with BC annually, most of whom have stage I (40.4%) or stage II (32.6%) disease, whereas 9.6% patients have stage III and 4.6% stage IV [2]. For stage IIB/III (advanced T-stage disease often with nodal involvement) or locoregional recurrent disease, curative treatment generally consists of surgery, radiotherapy and (neoadjuvant or adjuvant) systemic therapy (i.e., chemo-, endocrine and targeted therapy) [3]. In the case of metastatic disease without curative options, burdensome locoregional as well as systemic therapy should be avoided in order to maintain quality of life. On the other hand, identification of oligometastatic disease may improve the chance of (prolonged disease free) survival by including these sites in the local therapy plan [4]. Therefore, accurate pre-operative staging is essential to identify locoregionally affected lymph nodes and (distant) metastases, as it will affect treatment choices.

The initial work-up for BC includes physical examination, mammography, ultrasound and magnetic resonance imaging (MRI) of the breast and axilla, to assess the extent of locoregional disease [3]. Standard staging procedures detect (distant) metastases in approximately 7% and 8–21% of clinical stage IIB and III patients, respectively, and in 33% of those presenting with locoregional recurrences [5,6,7]. Furthermore, 10–25% will develop recurrences within 2 years, suggesting, at least in part, missed (occult) metastases at presentation [8]. According to (inter)national guidelines, staging is often performed with 2-[^18^F]fluoro-2-deoxy-D-glucose (FDG) positron emission tomography accompanied by a low-dose CT scan for attenuation correction (FDG PET) [3,9,10]. In addition to this, a diagnostic computed tomography (CT) scan of the thorax and abdomen is often performed [3,9]. For primary staging of clinical stage II/III BC, the sensitivity and specificity of FDG PET to identify lymph node involvement and distant metastases is 63–100% and 98–100%, respectively [5,11], and in recurrent disease, it is 90% and 81%, respectively [12].

However, FDG uptake of BC can be quite variable, due to various underlying biological features [11,12,13,14,15,16,17,18]. FDG uptake is often lower in lobular BC (vs. ductal BC) [15,16,18,19], in low-intermediate grade (vs. high grade) [12,13,14,15,16,17,18] tumors and in ER-positive tumors, compared to triple negative tumors (ER-/PR-/HER2-) [11,13,15,16,17,20,21,22,23]. Alternatively, triple negative BC (ER-/PR-/HER2-), a more aggressive phenotype, shows higher FDG uptake than ER+/PR+ and HER2- BC [20]. Thus, these biological factors can affect the FDG avidity of lesions potentially limiting the accuracy of FDG PET/CT for the staging of grade 1–2 ER+ BC [16].

Although there are data that FDG uptake (usually expressed as standard uptake value (SUV)) is lower in low grade ER+ BC than in other types of BC and that staging might be suboptimal [24,25], no study has specifically investigated the extent to which this affects the staging of BC. Therefore, the primary aim of this study was to retrospectively investigate the diagnostic performance of FDG PET in staging patients with grade 1–2 ER + BC. The secondary aims were to study whether the level of tracer uptake in the primary tumor was associated with the accuracy of staging, and to investigate which histopathological features might predict the accuracy of FDG PET.

## 2. Materials and Methods

### 2.1. Patient Population

In this retrospective study, we included women ≥18 years with histologically proven ER+, grade 1–2, clinical stage IIB/III or locoregional recurrent BC, treated at the Amsterdam UMC (VUmc) and The Netherlands Cancer Institute-Antoni van Leeuwenhoek (NKI-AvL) in the Netherlands between 2008–2016 and 2014–2015, respectively. All patients underwent FDG PET/CT for staging and had their follow-up visits for at least 18 months. Patients with other malignancies in the last five years prior to diagnosis of (recurrent) BC were excluded.

Prior to inclusion, patients provided written informed consent, except when it was not possible to approach them for consent, due to various reasons (e.g., death or no contact details available). The study was approved by the local Medical Ethics Committee of the VUmc (No. 2017.382). 

### 2.2. Imaging Procedures

According to the standard of care, patients underwent mammography, ultrasound and MRI of the breast and axilla for locoregional staging. Patients at VUmc underwent an additional diagnostic CT scan of the thorax/abdomen and at both centers bone scans were performed if indicated (i.e., ‘conventional imaging’). FDG PET scans were performed using Gemini TF-64 or Ingenuity TF-64 PET/CT scanner at VUmc and Gemini TF-16 or Gemini TF-Big Bore 16 (Philips Medical Systems, Cleveland, Ohio, United States of America) at AvL, according to the guidelines of the European Association of Nuclear Medicine (EANM) [26]. Patients were administered 3.5 MBq/kg FDG at VUmc, and 190–240 MBq (according to the body mass index) at AvL [26]. All patients underwent a low-dose CT scan for attenuation correction, followed by the PET scan (skull vertex to mid-thigh) at 60 min post-injection, with 2 min per bed position.

### 2.3. Histopathology 

According to standard of care, the biopsy of the primary tumor was used to evaluate the histological subtype, grade (according to the Bloom–Richardson grading system), ER, PR, HER2 expression and mitotic activity. Compliant with Dutch guidelines ER-/PR-positivity on immunohistochemistry (IHC) was established if ≥ 10% of cell nuclei were immunoreactive, and HER2 was classified positive with 3+ or 2+ and amplified [3]. Mitotic activity was defined as the number of mitoses per 2 mm^2^. Suspect locoregional or distant lesions visible on conventional imaging and/or FDG PET that were decisive for therapy choices were verified by core needle biopsy and/or fine-needle aspiration cytology.

The pathological reports of lymph node resection were classified as follows: in the case of the presence of malignant cells, pathologically verified malignant lymph node; in the case of fibrosis compatible with complete response after neo-adjuvant therapy, pathologically verified malignant lymph node before neo-adjuvant treatment; and in the case of no malignant cells or fibrosis by cytology and/or histology, benign lymph node.

### 2.4. Patient-Based Analysis

The patient-based outcomes consisted of determining the stage of disease at baseline and at the end of follow-up. The stage of disease was determined at clinical presentation together with conventional imaging and subsequently by FDG PET together with pathological confirmation. 

### 2.5. Lesional Analysis: Qualitative and Semi-Quantitative FDG PET Readings

Conventional imaging was performed ≤5 weeks before or after FDG PET. Clinically relevant lesions suspicious for malignancy on any imaging modality were included in this analysis, with a maximum of 5 largest lesions per tissue type in the case of distant metastases. The included lesions were either pathologically confirmed as benign or malignant (group A) or, in the case of absent/inconclusive pathology, verified by additional imaging and/or follow up for 18 months after primary diagnosis (group B). Based on these data, we classified lesions as true positives (=malignant lesions suspect on FDG PET), true negatives (=benign lesions not suspect on FDG PET), false positives (=benign lesions suspect on FDG PET) and false negatives (=malignant lesions not suspect on FDG PET). In the case of multiple axillary lymph nodes on FDG PET, only those which were pathologically proven to be malignant were included in group A. 

Quantitative analysis was performed, using in-house developed software (version 04092018, Accurate tool, R. Boellaard, Amsterdam, The Netherlands) [27]. This analysis only included lesions visible on FDG PET. Volumes of interest (VOIs) were semi-automatically defined using 50% thresholds of peak standardized uptake values (SUV_peak_) adapted for local background^26^ and verified by radiologists. For each VOI, we determined the SUV_max_, SUV_mean_, SUV_peak_ and total lesion glycolysis (TLG). In addition, for primary breast lesions, VOIs were manually defined on the low-dose CT scans to calculate anatomical volumes. The correlation between these FDG PET parameters and various histopathological features of the primary tumor was assessed to investigate whether histopathological features predicted the accuracy of the FDG PET.

### 2.6. Clinical Implications on Treatment Plan

We investigated the impact of incorrect lesion identification by FDG PET. The pathological outcome of the surgically resected axillary lymph nodes was retrospectively compared to lymph nodes identified on FDG PET, excluding patients that had progressive disease during neo-adjuvant therapy. We postulated that without progression, any additional pathologically verified malignant node in the resection specimen, compared to baseline FDG PET, should be classified as false negative on FDG PET. In the case that this would lead to stage migration of the N-stage, from N1 to N2, such patients would require an axillary lymph node dissection, according to current guidelines instead of sentinel node/marked node resection [3].

Similarly, the number of distant metastases is relevant for the treatment plan. In the case of oligometastatic disease (<4 lesions), local treatment with curative intent can be considered, whereas in the case of extensive disease (≥4 distant metastases) a palliative option will prevail. We compared the distant lesions on FDG PET to the number of distant metastases confirmed through pathological verification and/or additional imaging at baseline and during the follow-up period.

### 2.7. Statistical Analysis

Statistical analysis was performed, using SPSS Statistics 22.0 (IBM Corp.). Accuracy was measured at the patient and lesional levels, separately for groups A and B. The difference between FDG PET parameters (SUV_max_, SUV_peak_, SUV_mean_ and TLG) across the different categories in the two groups (true positives, false positives and false negatives, group A and B) was assessed using the Kruskal–Wallis test. The association between the tracer uptake in the primary tumor and accuracy of staging was assessed by using the Mann–Whitney U test. The association between histopathological features of the primary tumor and accuracy of staging was assessed by using a chi-square test or a Mann–Whitney U test. The association between semi-quantitative FDG PET parameters and histopathological features of the primary tumor was investigated, using a mixed model analysis with an intercept on patient level. Results were considered significant for a *p*-value of <0.05.

## 3. Results

### 3.1. Patients

Seventy-four patients (37 from each center) with a median age of 49 years (range: 28–94) were included. Most patients presented with clinical stage IIB (48.6%) or III (47.3%) BC (Table 1). FDG PET/CT was performed after primary surgery in four patients, and prior to surgery (*n* = 6) or systemic treatment (*n* = 58 neo-adjuvant and *n* = 6 palliative) in the remaining 70 patients.

### 3.2. Patient-Based Analysis 

In 67% (47/70), the FDG PET stage was identical to the clinical stage at baseline and in 10% (7/70), FDG PET correctly upstaged patients (Table 2). However, of the remaining 16 patients, 3 were incorrectly downstaged and 13 were incorrectly upstaged. Four patients underwent staging with FDG PET after surgery, as they had stage IIB or III disease post-surgery: one of these patients had an additional suspect breast lesion on FDG PET, and subsequent mastectomy showed multifocal breast cancer.

At the end of follow-up (after 18 months), 81.4% were disease-free (Table S1). Of 67 patients who were diagnosed with locoregional disease at baseline, 3 developed metastases during follow-up. One patient had stage III by FDG PET at baseline; during follow-up, multiple bone metastases were diagnosed after 12 months. The second patient had multiple FDG-avid mediastinal lymph nodes, which were classified as reactive lymph nodes (no biopsy/cytology performed), and 17 months later, she developed pathologically proven liver metastases (without growing mediastinal nodes). The third patient had enhanced uptake in parasternal and paratracheal lymph nodes (no biopsy/cytology performed), which was interpreted at baseline as reactive lymph nodes probably due to esophagitis; 9 months later she presented with mastitis carcinomatosa, growing parasternal lymph nodes and liver metastases.

### 3.3. Lesional Analysis

**Figure 1:**
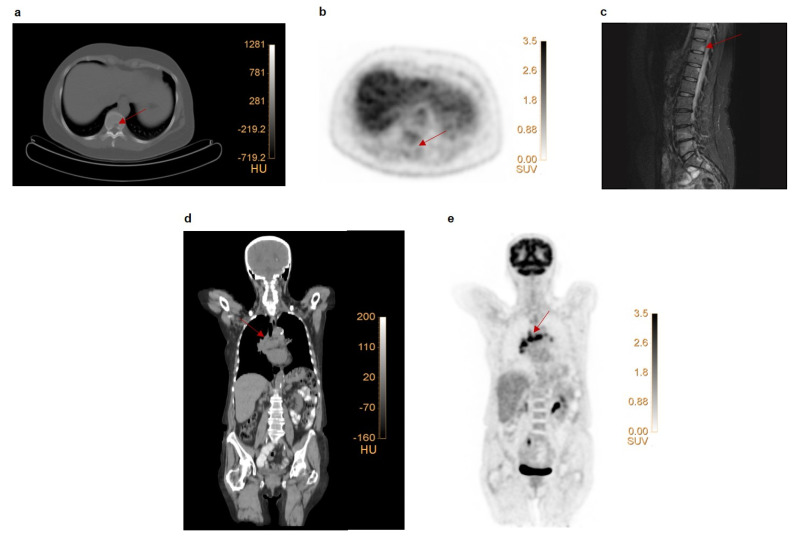
**Examples of false negative and false positive lesions on FDG PET.** (**a**–**c**) Patient with primary ER+ breast cancer with faint uptake in the primary tumor (SUV_max_ 2.3). Low-dose CT (**a**) revealed a lytic lesion in the 10th thoracic vertebra (Th10) without enhanced FDG uptake (**b**). An MRI scan (**c**) revealed multiple vertebral metastases (Th4, Th11, Th12, L4, L5), including the one at Th10. This lesion was classified as false negative on FDG PET. (**d**–**e**) Patient with multiple mediastinal FDG avid, suspect lymph nodes. Coronal section of a low-dose CT-scan (**d**) and FDG PET scan (**e**). Endobronchial ultrasound–guided biopsy of 3 mediastinal lymph nodes showed reactive cells. These lesions were, therefore, classified as false positive on FDG PET.

In group A, 155 lesions were pathologically verified prior to neo-adjuvant therapy and primary surgical treatment (breast: 86, locoregional lymph nodes: 58, distant: 11; Table S2A). Visual analysis of FDG PET correctly classified 115/155 (74.2%) lesions as malignant, and 16/155 (10.3%) as benign. FDG PET incorrectly categorized 24/155 (15.5%) lesions: 7/155 (4.5%) lesions in 7 patients were malignant but showed no uptake, whereas 17/155 (11.0%) lesions in 9 patients (5 with 1 lesion, 2 with 2 lesions, and 2 with 4 lesions) were benign but showed enhanced uptake (Figure 1). On this pathologically confirmed lesional basis, FDG PET had a sensitivity and specificity of 94.3% and 48.4%, respectively.

Group B consisted of 112 lesions (Table S2B). FDG PET classified 61/112 (54.5%) and 8/112 (7.2%) lesions as true positives and true negatives, respectively. Forty-three (43/112, 38.4%) lesions were classified incorrectly: 12/112 (10.8%) malignant lesions showed no uptake, whereas 31/112 (27.7%) lesions showed enhanced uptake reported as suspect but were benign. On this, with imaging/follow up confirmed lesional basis, FDG PET had a sensitivity and specificity of 83.6% and 20.5%, respectively.

Results of groups A and B taken together (Table S2C) yielded a sensitivity and specificity of 90.3% (95% CI 85.3–93.7%) and 33.3% (23.5–44.8%), respectively. Misclassification by FDG PET mostly involved axillary lymph nodes and bone tissue, respectively (Table S3).

A similar lesion-based analysis was performed for conventional imaging, including the diagnostic CT scan (Table S4), showing high sensitivity and low-moderate specificity rates of 95.9% and 15.2% and 80.8% and 66.7% for groups A and B, respectively. Outcomes of conventional and FDG PET imaging were also combined together for group A (Table S5), showing that conventional imaging alone identified 23 additional suspect lesions of which 7 were malignant. FDG PET alone identified 10 other suspect lesions of which 5 were malignant.

**Figure 2:**
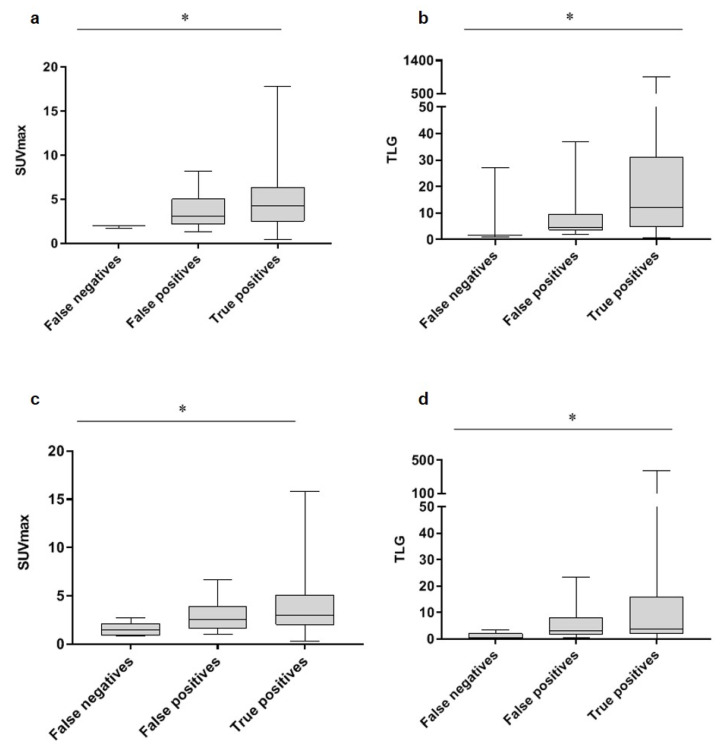
**SUV_max_ and TLG show no significant differences between false and true positive lesions**. Lesions were classified into 3 groups, i.e. false negatives, false positives and true positives; lesions have been verified with pathology (**a**,**b**) or additional imaging and/or follow-up (**c**,**d**). Similar results have been obtained for SUV_peak_ and SUV_mean_ (Supplementary Figures S1 and S2). * *p* < 0.05.

Quantification of visually identified lesions on FDG PET did not improve discrimination between true and false positives lesions (Figure 2, Table S6 and Figures S1–S3), in either group (A and B).

### 3.4. Correlation between FDG PET Parameters and Histopathology

FDG uptake in the primary tumor was not associated with the accuracy of FDG PET staging (*p* = 0.67). Ductal carcinoma had a higher SUV_peak_ and SUV_mean_ than lobular carcinoma (*p* < 0.05), and HER2+ tumors had a significantly higher TLG compared to HER2- tumors (*p* < 0.05) (Table S7). The % ER positivity correlated with TLG (*p* < 0.05)

### 3.5. Implications for the Plan

In summary, in 22/74 (29.8%) patients, the treatment plan based solely on FDG PET imaging would have been incorrect. In total, 65/74 (87.8%) patients underwent surgical resection, and in 34/65 patients (52.3%), surgery included also axillary lymph node dissection. No patient on neo-adjuvant therapy had progressive disease during treatment. Pathological analysis of axillary specimens classified 143 of 346 lymph nodes as malignant, whereas 203 were benign (Table S8). Since it is impossible to match each lymph node in the pathology specimen with their location on imaging, we compared the numbers of suspicious nodes on FDG PET with malignant nodes in the specimen. 83/143 (58.0%) malignant lymph nodes in 16 patients were classified as false negatives on FDG PET. In 7 patients, diagnosed with N1-stage disease on FDG PET, axillary lymph node dissection showed N2-disease (Table S9). In 2 patients with one malignant node on FDG PET, 1 or 2 additional nodes were identified when the axillary lymph node dissection was performed. Additionally, FDG PET falsely identified N3 disease (infraclavicular lymph node) in one patient, whereas in one case, N3 disease (intramammary lymph node) was missed. In the remaining patients, FDG PET showed the same number or fewer affected lymph nodes than the resection specimen, the latter most likely due to the effect of the neo-adjuvant systemic treatment. As the neo-adjuvant treatment affects the lesion size, no correlation between FDG PET positivity and the size of the lymph node metastasis could be made.

Metastatic disease was missed by FDG PET in two patients: one patient had multiple bone metastases and the other patient had a lung metastasis. In eight other patients, false positive lesions were identified in the liver, thyroid, bone and lymph nodes located in the neck, mediastinum and inguinal region (Table S3).

## 4. Discussion

To our knowledge, this is the first study assessing the diagnostic performance of FDG PET in patients with stage IIB/III or LRR, grade 1–2, ER+ breast cancer. In this study, the sensitivity of FDG PET for disease staging was 77.1%. Previous studies have reported a sensitivity of up to 100% for primary breast cancer [15,28] and 81–97% for restaging of LRR [15], for all types of breast cancer combined. In a meta-analysis performed by Han et al. [29], it could be seen that FDG PET outcomes led to changes in staging in 25% of patients. In addition, various studies have shown that FDG PET outcomes affected the treatment plan in 6.5–18% of patients with primary breast cancer [11,15,29]. These data reinforce the importance of additional imaging modalities next to the conventional imaging to obtain the correct stage, which is essential for an adequate treatment plan. In our case, the treatment plan was correctly adapted by FDG PET in 7/70 (10%) patients, but in 16/70 (23%) patients FDG PET would have led to an incorrect treatment plan (Table 2). Thus, our results support the hypothesis that FDG PET is insufficient for (re)staging of grade 1-2 ER+ breast cancer. 

### 4.1. TNM Lesion Detection

When looking into more detail to detection of individual lesions, this study shows that the sensitivity and specificity of FDG PET for lesion detection (pre-operatively) was 94.3% and 48.8% (group A)/83.6% and 20.5% (group B), respectively in patients with grade 1–2, ER+ BC. Differentiation of lesion detection based on the type of lesion (i.e., primary breast lesions, locoregional lymph nodes and distant metastases), showed that FDG PET accurately detects primary breast tumors (83/87 (95.4%). Our data are in line with a prospective study that showed similar detection rate of BC lesions when comparing FDG PET/CT with MRI (95% vs. 100%, *p* = 1.0) [30]. However, compared to other conventional imaging techniques, such as MRI, it is known that FDG PET has less sensitivity and less accuracy for determining the size of the tumor and to assess the presence of multifocal disease [15]. For locoregional lymph nodes, previous studies have shown that micrometastases are suboptimally detected with FDG PET (CT) [31,32]. However, in current clinical practice, it is essential to identify all affected nodes before neo-adjuvant treatment, as only extensively affected axillary lymph nodes (i.e., ≥N2-disease/‘bulky’ disease) remaining after neo-adjuvant systemic treatment will, in general, require axillary lymph node dissection. In the case of N1-disease (1–≤ 3 affected lymph nodes) at diagnosis and response to neo-adjuvant treatment, resection of the sentinel node(s) and marked node is deemed sufficient when followed by locoregional radiotherapy [3]. In our study, 26/96 (27.1%) axillary lymph nodes were incorrectly identified: 3.1% of the axillary lymph nodes were identified as false negatives and 24.0% as false positive nodes (Table S3). These incorrect identified nodes could potentially change the N-stage and eventually the locoregional treatment, making it even more important that these nodes are correctly identified.

In the case of distant metastases, FDG PET(CT) is known to have a high yield as shown in inflammatory and stage II/III BC [6,15,33,34,35]. In this study, distant metastases were identified in 7/70 patients (10%), which is at the lower end of what would be expected from the literature for stages IIB/III/LRR [5,6,7]. In 4 patients, FDG PET confirmed the suspicion of metastases as seen on conventional imaging, and in 3 patients, metastatic lesions were correctly identified on FDG PET alone. However, FDG PET also missed lung and bone metastases in 7 patients. Distant metastases were mainly located in extra-axillary lymph nodes, the lungs and bone. FDG PET lacks sensitivity for the detection of (small) lung nodules (due to partial volume effect and respiratory movement) and identifies osteoblastic lesions suboptimally (often showing low or no FDG uptake in these lesions) [15,36]. In our study, most of the lung lesions were small (range: 4–11 mm) and therefore correct identification of these might have been hampered by the partial volume effect; however, the low grade, ER+ breast cancer subtype might also have played a role. Most of the bone lesions included in this study were osteolytic and also for these lesions, applied that the specified low grade, ER+ breast cancer subtype might have affected its identification on PET. 

### 4.2. Association between FDG PET Parameters and Histopathology

Quantification of FDG uptake only showed a trend for higher SUV_max_ and TLG values in malignant (true positive) lesions, compared to false positives and false negatives. However, no specific threshold for malignancy could be determined, as was described in other studies [37,38]. The histological subtype, however, correlates with FDG uptake, with ductal BC having higher FDG uptake, compared to lobular BC. This is in accordance with other studies and can probably be explained by a lower tumor cell density, a low level of GLUT1 expression, diffuse infiltration of surrounding tissue and a decreased proliferation rate in lobular BC, eventually resulting in lower FDG uptake [17,19,20].

We did not observe a difference in FDG uptake between grade 1 and grade 2 tumors. In the literature, it is known that grade 3 tumors have significantly higher FDG uptake than grade 1–2 tumors, but no information is available regarding the correlation between FDG uptake and grade 1 and 2 tumors, separately [12,13,14,15,16,17,18]. Regarding the receptor status, we found that the % ER positivity and HER2 status correlated with TLG. No correlations could be found between the PR status and FDG uptake. Previous studies are somewhat contradictory about this: a few studies have shown that there is no correlation between the hormone receptor status (positive or negative) and FDG uptake [14,39], whereas others have shown that FDG uptake is affected by hormone receptor status [13,15,16,17,20,21,22]. These studies do not take the expression levels of ER and PR, separately and in combination, into account, which may be essential to identify the relation with FDG uptake. For HER2, no correlation could be found between its status (positive/negative) and FDG SUV, which is consistent with other studies [16,17,22]. However, the HER2 status did correlate with TLG, but we could not confirm this from other studies, as they did not include TLG in their analyses. 

We did not find a correlation between the FDG uptake and the mitotic activity index (mean ± standard deviation: 3.1 (±4.1); range: 0–19), probably as we only included tumors, which are expected to be less metabolically active than other subtypes of breast cancer. Studies including more metabolically active tumors, identified by a higher Ki-67 expression, have reported higher FDG uptake [17,40].

### 4.3. Limitations

Due to the retrospective set-up of this study not all clinical, imaging, and pathology data were available for all patients. We had access to all the FDG PET scans, but the scans of other imaging modalities were not always present. In those cases, the available report of the radiologist was used to compare lesions on the different imaging modalities. Furthermore, of the 267 lesions investigated, 155/267 lesions were pathologically verified (reference method), whereas for 112/267 lesions, only additional imaging and/or follow-up data were available, which precludes a definitive diagnosis regarding these lesions. However, our separate analyses for both groups yielded results in a similar range, supporting the chosen approach. For the visual analysis, we also included all axillary lymph nodes, benign or malignant, as verified according to the pathology report. However, in the case of multiple avid lymph nodes on the FDG PET scan, it can be difficult to match the exact lymph node that was pathologically proven benign or malignant to the correct lesion on the scan. In that case, it was assumed that the lesion that is most avid on the scan is also most likely the one of which biopsy or cytology is performed, and this lesion could also be quantified. Of the remaining lesions, only the number of affected lymph nodes were taken into account in the analysis.

### 4.4. Scientific Implications

Imaging with FDG PET for patients with grade 1–2, ER + BC can potentially lead to incorrect staging. In the search for alternative methods to improve staging, imaging based on the ER, which is independent of metabolic activity, might be of interest. Several clinical studies have shown that 16α-[^18^F]-fluoro-17β-estradiol ([^18^F]FES) PET/CT has overall high sensitivity (82–84%) and specificity (93–98%) rates [41], making it an interesting ER-targeting PET tracer to compare with FDG for the staging of patients with grade 1–2, ER + BC.

## 5. Conclusions

The data presented in this study show that FDG PET imaging inadequately staged 22.9% of grade 1–2, ER+ BC cases. This can lead to incorrect staging and subsequently to inappropriate treatment choices, potentially affecting survival and quality of life. Prospective studies investigating novel radiotracers are urgently needed to improve the current imaging staging procedures.

## Figures and Tables

**Table 1:** Patient characteristics.

	**N (%) or Median (Range)**
**Age at diagnosis (y)**	49 (28–94)
**Clinical stage at presentation**	
IIB	36 (48.6)
III	35 (47.3)
Locoregional recurrence	3 (4.1)
Histological subtype *	
Ductal	57 (77.0)
Lobular	17 (22.7)
Micropapillary	1 (1.3)
**Grade**	
1	7 (9.5)
2	67 (91.5)
**ER receptor**	
Positive	74 (100.0)
**PR receptor**	
Negative	13 (17.6)
Positive	61 (82.4)
**HER2neu receptor**	
Negative	65 (87.8)
Positive	9 (12.2)
**Treatment received**	
**Neo-adjuvant therapy**	
(after FDG PET imaging)	
yes	58 (78.4)
- chemotherapy	- 53 (91.4)
- endocrine therapy	- 5 (8.6)
no **	16 (21.6)
**Surgery**	
- yes	65 (87.8)
- before FDG PET imaging	- 4 (6.2)
- after FDG PET imaging	- 61 (93.8)
- no	9 (12.2)
**Adjuvant therapy**	
- yes	69 (93.2)
- no	2 (2.7)
- unknown	3 (4.1)

* One patient with multifocal BC presented with 2 lesions in the breast, each having a different histological subtype. ** These patients directly underwent surgery before (*n* = 4) or after the FDG PET scan (*n* = 6) or received endocrine treatment for metastatic disease (*n* = 5) or locoregional recurrence (*n* = 1) after FDG PET imaging.

**Table 2:** **FDG PET staged 16/70 patients incorrectly compared to final baseline stage.** Patients who received FDG PET imaging prior to surgery or who did not receive surgical treatment are included (*n* = 70). The table indicates the number of patients with their corresponding stage. (A) Staging based on clinical assessment, pathology and conventional imaging performed at baseline. (B) Staging based on FDG PET imaging. (C) Final stage determined after FDG PET imaging, additional imaging and/or biopsy/cytology of new identified suspect lesions.

(A) Clinical Stage	(B) [^18^F]FDG PET Stage	(C) Final Stage Baseline			
		Local Recurrence	IIB	III	IV
**Local recurrence**	**Local recurrence**	2	0	0	0
	**IIB**	0	0	0	0
	**III**	0	0	0	0
	**IV**	0	0	0	1
**IIB**	**Local recurrence/stage I/IIA**	0	0	0	0
	**IIB**	0	21	0	0
	**III**	0	3	0	0
	**IV**	0	4	0	1
	**No lesions visible on scan**	0	1	0	0
**III**	**Local recurrence/stage I/IIA**	0	0	0	0
	**IIB**	0	0	2	0
	**III**	0	0	24	0
	**IV**	0	0	6	5

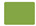
: Correctly identified by FDG PET, 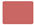: Incorrectly downstaged by FDG PET, 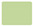: Correctly upstaged by FDG PET, 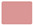: Incorrectly upstaged by FDG PET.

## Data Availability

The data supporting the conclusions of this article are included within the article.

## Supplementary Materials

The following are available online at https://www.mdpi.com/article/10.3390/diagnostics11111954/s1. Please see ‘Supplemental data’.
