# Supplementary material for: Diagnostic Performance of [18F]FDG PET in Staging Grade 1–2, Estrogen Receptor Positive Breast Cancer
Source: Diagnostics (Basel). 2021 Oct 21;11(11):1954. doi: 10.3390/diagnostics11111954 (PMC8625348; doi:10.3390/diagnostics11111954)
Supplement: Supplementary file 1 [file diagnostics-11-01954-s001.zip › diagnostics-1394885-supplementary.pdf]

Appendix A: Supplementary data

Table S1. Clinical outcome: 3 out 67 patients with local disease at baseline had progressive disease during follow-up. Follow-up data of 18 months was available for 70 out of 74 patients.

	Clinical outcome at the end of follow-up *(n = 70)*				
**Final stage at baseline**	**Stage at end of follow-up**	**Cured or stable disease N (% of total)**	**Progressive disease N (% of total)**	**Death caused by breast cancer N (% of total)**	**Death due to other cause N (% of total)**
**Local recurrence**	M0	1 (1.4)	0 (0.0)	0 (0.0)	1 (1.4)
	M1	0 (0.0)	0 (0.0)	0 (0.0)	0 (0.0)
**IIB**	M0	31 (44.3)	1 (1.4)	0 (0.0)	0 (0.0)
	M1	0 (0.0)	0 (0.0)	0 (0.0)	0 (0.0)
**III**	M0	25 (35.7)	0 (0.0)	0 (0.0)	1 (1.4)
	M1	0 (0.0)	2 (2.9)	1 (1.4)	0 (0.0)
**IV**	M0	0 (0.0)	0 (0.0)	0 (0.0)	0 (0.0)
	M1	3 (4.3)	3 (4.3)	1 (1.4)	0 (0.0)

Table S2. On a lesion-basis FDG PET incorrectly classified 24 of 287 pathologically verified lesions (A) and 43 of 204 lesions verified by follow up (B). Classification of lesions into 4 groups, based on the visual analysis using FDG PET imaging. Lesions have been pathologically verified (table A) or in case of absent/inconclusive pathology been verified by additional imaging and/or follow-up (table B). Outcomes of table A and B have been taken together in Table C.

A

			**Pathologically verified lesions**		
			**Malignant (% of all total lesions)**	**Benign (% of all total lesions)**	**Total**
**FDG PET**	**Uptake present and suspect**				
		Breast	72	1	73
		Locoregional lymph nodes	41	9	50
		Distant	2	7	9
		**Total**	**115 (74.2)**	**17 (11.0)**	**132**
	**No uptake and not suspect**				
		Breast	3	10	13
		Locoregional lymph nodes	3	5	8
		Distant	1	1	2
		**Total**	**7 (4.5)**	**16 (10.3)**	**23**
		**Total of all lesions**	**122**	**33**	**155**

B

			Lesions verified with additional imaging and/or follow-up		
			**Malignant (% of all total lesions)**	**Benign (% of all total lesions)**	**Total**
**FDG PET**	**Uptake present and suspect**				
		Breast	4	0	4
		Locoregional lymph nodes	33	11	44
		Distant lesions	24	20	44
		**Total**	**61 (54.5)**	**31 (27.7)**	**92**
	**No uptake and not suspect**				
		Breast	0	1	1
		Locoregional lymph nodes	2	0	2
		Distant lesions	10	7	17
		**Total**	**12 (10.8)**	**8 (7.2)**	**20**
		**Total of all lesions**	**73**	**39**	**112**

C

FDG PET			Lesion verification through pathology report, additional imaging and/or follow-up		
			**Malignant (% of all total lesions)**	**Benign (% of all total lesions)**	**Total**
	**Uptake present and suspect**				
		Breast	76	1	77
		Locoregional lymph nodes	74	20	94
		Distant	26	27	53
		**Total**	**176**	**48**	**224**
	**No uptake and not suspect**				
		Breast	3	11	14
		Locoregional lymph nodes	5	5	10
		Distant	11	8	19
		**Total**	**19**	**24**	**43**
		**Total of all lesions**	**195**	**72**	**267**

Correctly identified by FDG PET (green)

Incorrectly identified by FDG PET (red)

Table S3. Often incorrect identified lesions by FDG PET concerned axillary lymph nodes. Classification of lesions based on their location. All lesions have been verified with pathology, additional imaging and/or follow-up.

		Lesions verified with pathology, additional imaging and/or follow-up			
		**True negatives, n (% of total)**	**False negatives, n (% of total)**	**True positives, n (% of total)**	**False positives, n (% of total)**
**Location**	**Breast**	**11 (4.1)**	**3 (1.1)**	**76 (28.5)**	**1 (0.4)**
	**Axillary lymph nodes**	**6 (2.2)**	**3 (1.1)**	**64 (24.0)**	**23 (8.6)**
	**Other lymph nodes**				
	- neck	0 (0.0)	0 (0.0)	0 (0.0)	**2 (0.7)**
	- intramammary	0 (0.0)	**1 (0.4)**	0 (0.0)	0 (0.0)
	- parasternal	0 (0.0)	0 (0.0)	**3 (1.1)**	0 (0.0)
	- infraclavicular	0 (0.0)	**1 (0.4)**	**5 (1.9)**	**2 (0.7)**
	- supraclavicular	0 (0.0)	0 (0.0)	**2 (0.7)**	0 (0.0)
	- retropectoral	0 (0.0)	0 (0.0)	0 (0.0)	**1 (0.4)**
	- mediastinal	0 (0.0)	0 (0.0)	**9 (3.4)**	**7 (2.6)**
	- aorta	0 (0.0)	0 (0.0)	0 (0.0)	**5 (1.9)**
	- hilus lung	0 (0.0)	0 (0.0)	**1 (0.4)**	**1 (0.4)**
	- hilus liver	0 (0.0)	0 (0.0)	0 (0.0)	**1 (0.4)**
	- inguinal	0 (0.0)	0 (0.0)	0 (0.0)	**1 (0.4)**
	**Thyroid**	0 (0.0)	0 (0.0)	0 (0.0)	**1 (0.4)**
	**Lung**	**7 (2.6)**	**4 (1.5)**	0 (0.0)	0 (0.0)
	**Liver**	0 (0.0)	0 (0.0)	0 (0.0)	**2 (0.7)**
	**Bone**	0 (0.0)	**7 (2.6)**	**16 (6.0)**	**1 (0.4)**
	**Total analyzed lesions**	**24**	**19**	**176**	**48**

Table S4. Conventional imaging incorrectly classified 33 out of 290 pathologically verified lesions (A) and 27 out of 184 lesions verified by follow up (B). Classification of lesions into 4 groups, based on the visual analysis using conventional imaging. Lesions have been pathologically verified (table A) or in case of absent/inconclusive pathology been verified by additional imaging and/or follow-up (table B).

A

Conventional imaging			**Pathologically verified lesions**		
			**Malignant (% of all total lesions)**	**Benign (% of all total lesions)**	**Total**
	**Suspect**				
		Breast	73	11	84
		Locoregional lymph nodes	41	11	52
		Distant	3	6	9
		**Total**	**117 (74.5)**	**28 (18.1)**	**145**
	**Not suspect**				
		Breast	2	0	2
		Locoregional lymph nodes	3	3	6
		Distant	0	2	2
		**Total**	**5 (3.2)**	**5 (3.2)**	**10**
		**Total of all lesions**	**122**	**33**	**155**

B

Conventional imaging			Lesions verified with additional imaging and/or follow-up		
			**Malignant (% of all total lesions)**	**Benign (% of all total lesions)**	**Total**
	**Suspect**				
		Breast	4	1	5
		Locoregional lymph nodes	25	1	26
		Distant lesions	30	11	41
		**Total**	**59 (52.7)**	**13 (11.6)**	**72**
	**Not suspect**				
		Breast	0	0	0
		Locoregional lymph nodes	10	10	20
		Distant lesions	4	16	20
		**Total**	**14 (12.5)**	**26 (23.2)**	**40**
		**Total of all lesions**	**73**	**39**	**112**

Green: Correctly identified by conventional imaging

Red: Incorrectly identified by conventional imaging

Table S5. Conventional imaging and FDG PET were concordant for 110 pathologically verified malignant lesions, were FDG PET identified 5 and conventional imaging 7 additional malignant lesions. Overview of pathologically verified lesions classified into 4 groups based on their outcomes on conventional and FDG PET imaging: not suspect-uptake, suspect-no uptake and suspect-uptake. There were no lesions with pathological verification for the group not suspect-no uptake.

Location of lesions	Pathologically verified lesions	
	Malignant (% of all total lesions)	Benign (% of all total lesions)
	Conventional imaging: not suspect FDG PET: uptake	
Breast	2	0
Locoregional lymph nodes	3	3
Distant	0	2
Total	5 (3.2)	5 (3.2)
	Conventional imaging: suspect FDG PET: no uptake	
Breast	3	10
Locoregional lymph nodes	3	5
Distant	1	1
Total	7 (4.5)	16 (10.3)
	Conventional imaging: suspect FDG PET: uptake	
Breast	70	1
Locoregional lymph nodes	38	6
Distant	2	5
Total	110 (71.0)	12 (7.7)
Total of all lesions	122	33

Table S6. True and false positive lesions on FDG PET could not be discriminated based on SUV values. Semi-quantitative PET parameters for lesions classified into 3 groups, i.e. true positives, false positives and false negatives. Included lesions have either been pathologically verified (part A) or with additional imaging and/or follow-up (part B).

A

	PET parameters			
	**SUVmax***	**SUVpeak***	**SUVmean***	**TLG***
**False negatives** ***(n = 3)***	1.95 (1.71-1.98)	1.68 (1.18-1.87)	1.32 (1.22-1.43)	1.64 (0.92-27.12)
**False positives** ***(n = 17)***	3.07 (2.14-4.58)	2.27 (1.62-3.71)	2.04 (1.51-3.14)	4.62 (3.80-7.69)
**True positives** ***(n = 113)***	4.23 (2.54-6.37)	3.01 (1.89-4.49)	2.55 (1.54-3.42)	12.15 (4.75-31.10)

* Median values with interquartile range

	PET parameters			
	**SUVmax***	**SUVpeak***	**SUVmean***	**TLG***
**False negatives** ***(n = 5)***	1.44 (0.90-1.52)	1.14 (0.90-1.39)	1.11 (0.84-1.23)	0.33 (0.24-0.71)
**False positives** ***(n =30)***	2.51 (1.68-3.85)	1.83 (1.28-2.93)	1.70 (1.23-2.78)	3.19 (1.78-8.03)
**True positives** ***(n = 60)***	3.00 (2.07-5.03)	2.18 (1.32-3.76)	2.11 (1.41-3.25)	3.60 (2.05-15.22)

* Median values with interquartile range

Table S7. Histological subtype is the most prominent pathological feature correlating with FDG uptake, with ductal BC having higher FDG uptake compared to lobular BC. Correlation between PET parameters and histopathological features of the primary breast lesions as assessed with mixed model analysis.

	**PET parameters**				
	**SUVmax***	**SUVpeak***	**SUVmean***	**TLG***	**Anatomic volume***
**Histological subtype**					
Lobular	3.20 (1.98-4.27)	2.53 (1.63-2.75)	1.92 (1.12-2.35)	19.83 (7.52-31.10)	13.12 (3.07-27.33)
Ductal	5.18 (3.59-7.01)	3.75 (2.70-5.53)	2.84 (2.14-4.05)	18.10 (10.44-35.85)	7.87 (4.83-14.74)
Estimate (CI)	2.99 (-0.13-6.10)	2.39 (0.02-4.76)	1.89 (0.10-3.68)	0.78 (-15.64-17.20)	-8.97 (-15.07- -2.87)
p-value	0.060	**0.048**	**0.039**	0.923	**0.005**
**Grade**					
1	4.12 (3.82-4.71)	2.89 (2.77-3.55)	2.40 (1.87-2.66)	8.83 (7.83-18.28)	5.44 (4.03-8.87)
2	4.53 (3.13-6.85)	3.30 (2.31-4.76)	2.71 (1.96-3.65)	19.29 (11.27-36.36)	8.00 (4.53-18.94)
Estimate (CI)	0.05 (-3.80-3.91)	0.95 (-1.58-3.48)	0.87 (-1.04-2.78)	8.19 (-9.35-25.73)	3.88 (-2.63-10.40)
p-value	0.977	0.449	0.359	0.348	0.233
**ER positivity (%)**					
Estimate (CI)	0.06 (-0.04-0.16)	0.04 (-0.10-0.18)	0.04 (-0.06-0.14)	0.96 (0.01-1.90)	0.08 (-0.28-0.43)
p-value	0.231	0.569	0.450	**0.048**	0.660
**PR status**					
Negative	5.07 (4.08-7.67)	3.76 (2.72-6.11)	2.71 (2.50-4.76)	40.29 (23.19-111.52)	19.65 (8.06-83.90)
Positive	4.42 (3.13-6.42)	3.01 (2.24-4.75)	2.55 (1.88-3.55)	15.60 (9.37-32.92)	7.81 (4.10-14.39)
Estimate (CI)	-1.37 (-4.43-1.68)	-1.00 (-3.41-1.40)	-0.83 (-2.64-0.98)	-13.26 (-29.91-3.40)	-2.83 (-9.02-3.35)
p-value	0.366	0.401	0.358	0.115	0.357
**HER2neu status**					
Negative	4.23 (3.13-6.37)	3.02 (2.31-4.47)	2.55 (1.96-3.38)	17.89 (9.52-35.33)	7.94 (4.10-15.81)
Positive	5.74 (4.53-9.99)	4.56 (2.68-7.34)	3.32 (1.87-5.09)	30.99 (20.87-72.72)	9.38 (7.90-23.49)
Estimate (CI)	1.34 (-3.05-5.73)	1.38 (-1.93-4.68)	0.74 (-1.75-3.23)	27.71 (4.81-50.60)	4.20 (-4.31-12.70)
p-value	0.538	0.402	0.549	**0.019**	0.321
**Mitotic activity index**					
Estimate (CI)	-0.15 (-0.44-0.14)	-0.16 (-0.38-0.06)	-0.12 (-0.28-0.05)	-0.99 (-2.52-0.54)	-0.075 (-0.64-0.49)
p-value	0.291	0.143	0.161	0.198	0.789
**Tumor size**					
Estimate (CI)	-0.14 (-0.56-0.29)	-0.18 (-0.71-0.36)	-0.14 (-0.54-0.27)	4.29 (0.60-7.98)	1.66 (0.29-3.03)
p-value	0.518	0.506	0.495	**0.024**	**0.019**

* Median values with interquartile range

Table S8. FDG PET imaging correctly identified 256 out of 346 axillary lymph nodes. Classification of axillary lymph nodes identified after surgical resection, based on the visual analysis comparing outcomes of FDG PET imaging with pathology outcomes. All included lymph nodes have been pathologically verified. Presence of malignant cells or pathological signs of complete response after neo-adjuvant therapy classified a node as malignant.

	**Pathologically verified lesions**	
**FDG PET imaging**	**Benign (% of all total lesions)**	**Malignant (% of all total lesions)**
**Not suspect**	196 (56.6)	83 (24.0)
**Suspect**	7 (2.0)	60 (17.3)
**Total**	203	143

Table S9. In 7 patients FDG PET imaging did not correctly identify the N-stage which could potentially affect treatment choices. Comparison of suspicious axillary lymph nodes present on FDG PET with affected axillary lymph nodes present in the pathology specimen.

**FDG PET imaging** *Number of nodes*	**Axillary lymph node dissection** *Number of nodes*	**Number of patients**
1	2-3	2
1-3	≥4	7

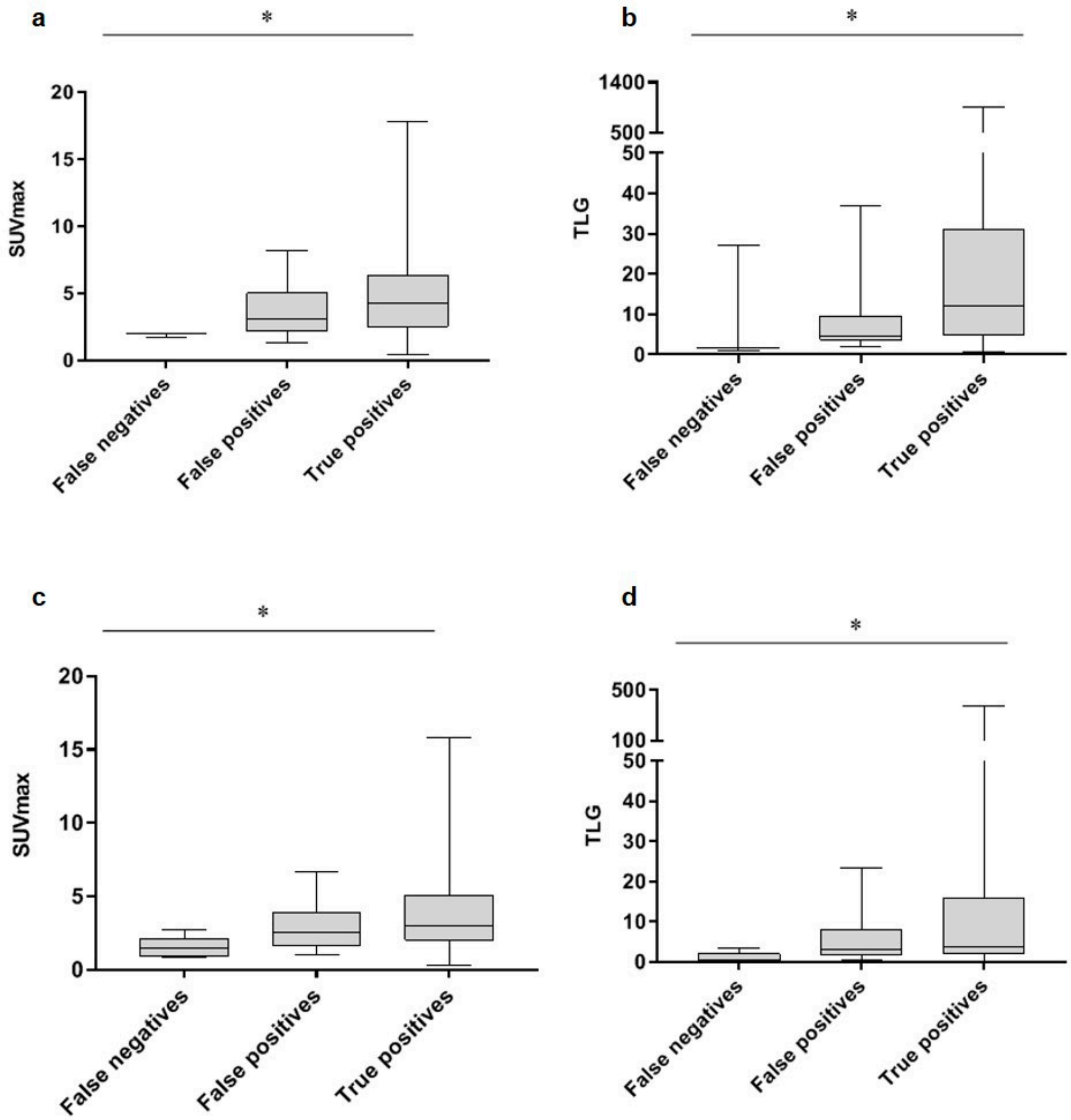

Figure S1. SUV_{max} and TLG show no significant differences between false and true positive lesions. Lesions were classified into 3 groups, i.e. false negatives, false positives and true positives; lesions have been verified with pathology (a/b) or additional imaging and/or follow-up (c/d). Similar results have been obtained for SUV_{peak} and SUV_{mean} (Suppl. Fig. 1 and 2). *P <0.05.

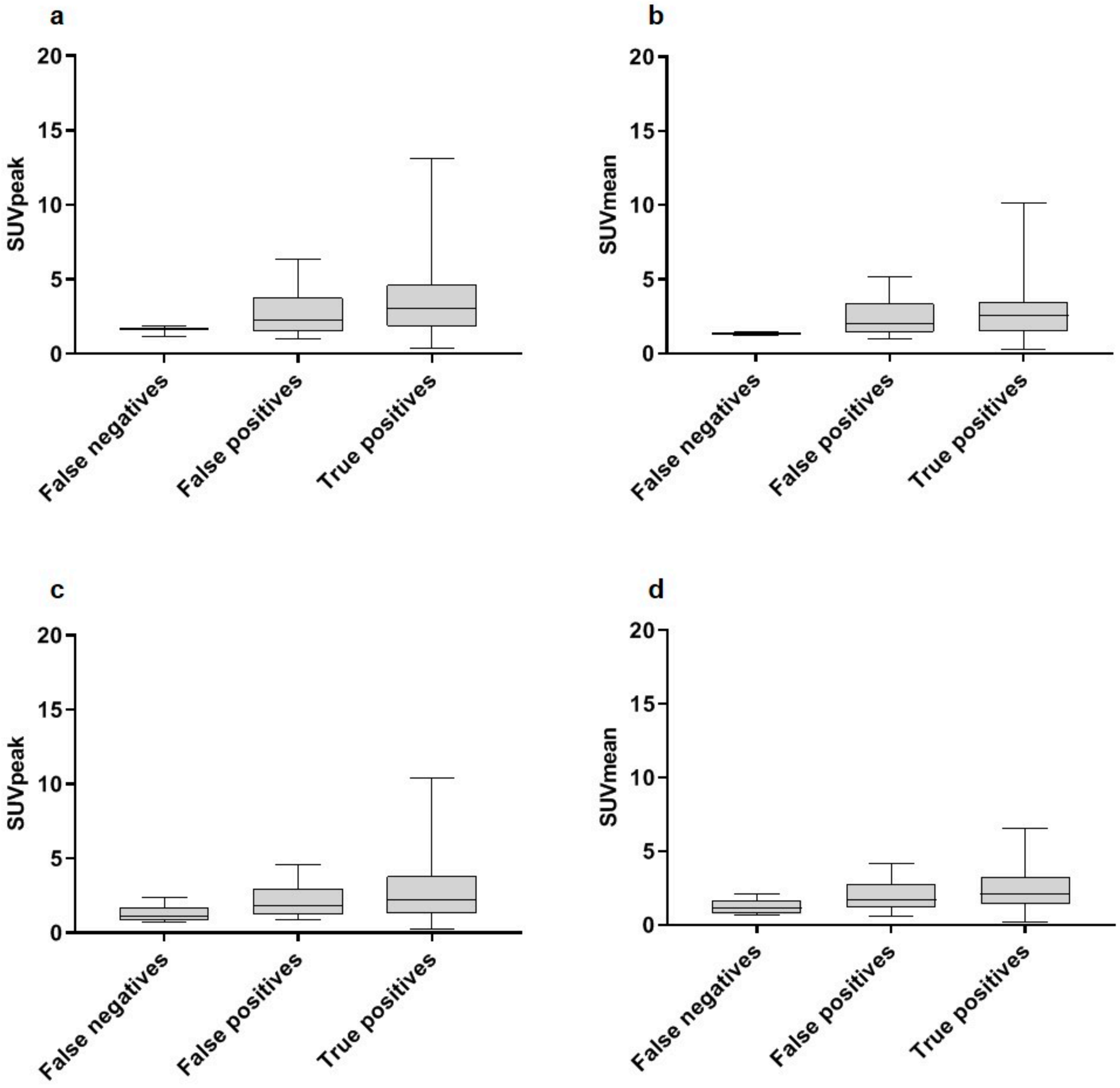

Figure S2. Semi-quantitative PET parameters (SUV_{peak} and SUV_{mean}) for lesions classified into 3 groups, i.e. false negatives, false positives and true positives). Lesions have been verified pathologically (a/b) or by additional imaging and/or follow-up (c/d).

$^{*}P < 0.05$

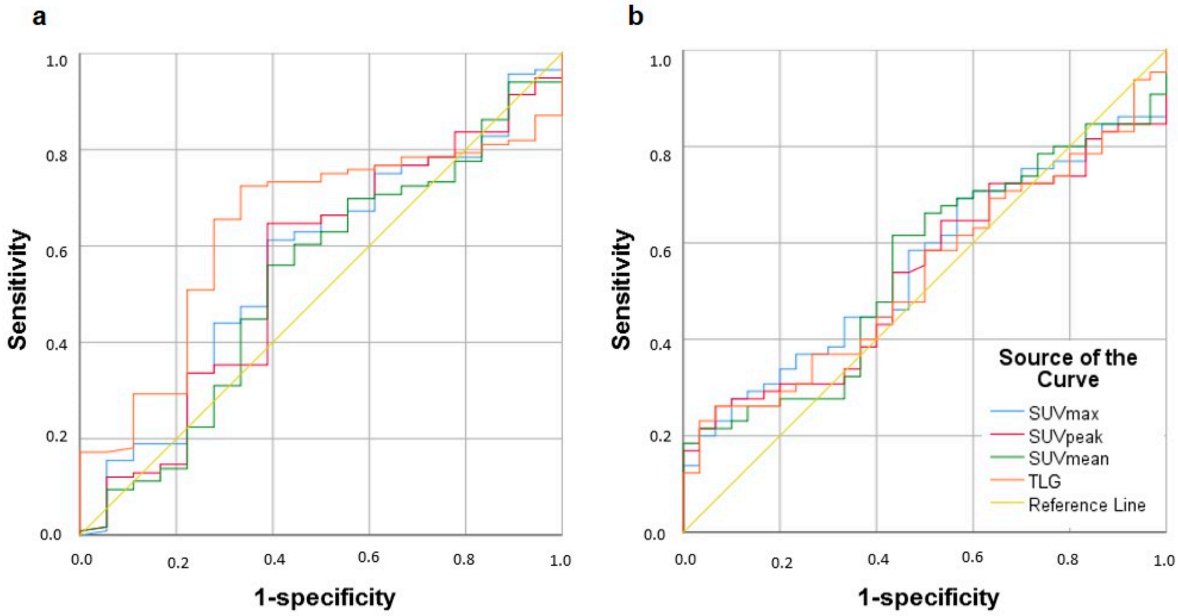

Figure S3. Receiver operating characteristics (ROC) curves for differentiation between malignant and benign lesions using the semi-quantitative PET parameters. Included lesions have either been pathologically verified (a) or with additional imaging and/or follow-up (b).
